# 2,4,6-Tris(2,4-dimethyl­phen­yl)-1,3,5-triazine

**DOI:** 10.1107/S1600536812016261

**Published:** 2012-04-21

**Authors:** Jin-Sheng Huang, Mao-Kui Li, Yang-Yi Yang, Seik Weng Ng, Edward R. T. Tiekink

**Affiliations:** a School of Chemistry and Chemical Engineering, Sun Yat-sen University, Guangzhou 510275, People’s Republic of China; b Department of Chemistry, University of Malaya, 50603 Kuala Lumpur, Malaysia; c Chemistry Department, Faculty of Science, King Abdulaziz University, PO Box 80203 Jeddah, Saudi Arabia

## Abstract

Two virtually superimposable mol­ecules comprise the asymmetric unit of the title compound, C_27_H_27_N_3_. The range of dihedral angles between the central 1,3,5-triazine ring and the attached benzene rings is 20.88 (14)–31.36 (14)°, and the shape of each mol­ecule is of a flattened bowl. The crystal packing features weak C—H⋯π bonds and π–π inter­actions between triazine and benzene rings [centroid–centroid separations = 3.7696 (17) and 3.7800 (18) Å] that result in the formation of supra­molecular layers in the *ac* plane. The crystal studied was a non-merohedral twin with a minor twin component of 20.7 (3)%.

## Related literature

For the synthesis, see: Orban *et al.* (1988 ▶). For the crystal structure of *s*-triphenyl­triazine, see: Damiani *et al.* (1965 ▶). For homologues, see: Bosch & Barnes (2002 ▶); Thalladi *et al.* (1999 ▶); Volkis *et al.* (2003 ▶). For the separation of twinned diffraction indices, see: Spek (2009 ▶).
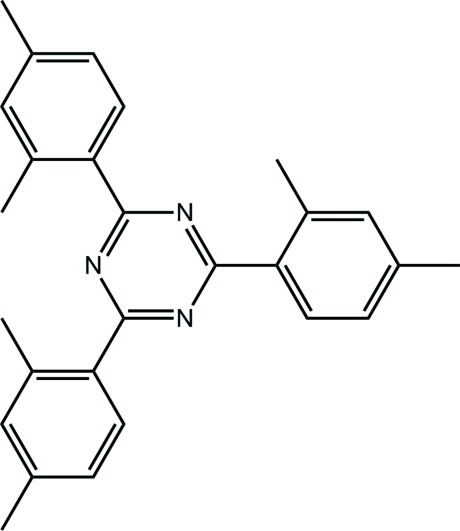


## Experimental

### 

#### Crystal data

C_27_H_27_N_3_
*M* *_r_* = 393.52
Triclinic, 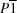

*a* = 7.4663 (4) Å
*b* = 15.0789 (13) Å
*c* = 19.7266 (12) Å
α = 109.016 (7)°
β = 90.949 (5)°
γ = 93.717 (6)°
*V* = 2093.6 (2) Å^3^
*Z* = 4
Mo *K*α radiation
μ = 0.07 mm^−1^
*T* = 100 K
0.30 × 0.15 × 0.05 mm

#### Data collection

Agilent SuperNova Dual diffractometer with an Atlas detector
Absorption correction: multi-scan (*CrysAlis PRO*; Agilent, 2012 ▶) *T*_min_ = 0.978, *T*_max_ = 0.996
14777 measured reflections
9605 independent reflections
5518 reflections with *I* > 2σ(*I*)
*R*_int_ = 0.058

#### Refinement

*R*[*F* ^2^ > 2σ(*F* ^2^)] = 0.085
*wR*(*F* ^2^) = 0.222
*S* = 1.04
9597 reflections
554 parameters
H-atom parameters constrained
Δρ_max_ = 0.36 e Å^−3^
Δρ_min_ = −0.39 e Å^−3^

### 

Data collection: *CrysAlis PRO* (Agilent, 2012 ▶); cell refinement: *CrysAlis PRO*; data reduction: *CrysAlis PRO*; program(s) used to solve structure: *SHELXS97* (Sheldrick, 2008 ▶); program(s) used to refine structure: *SHELXL97* (Sheldrick, 2008 ▶); molecular graphics: *X-SEED* (Barbour, 2001 ▶), *Qmol* (Gans & Shalloway, 2001 ▶) and *DIAMOND* (Brandenburg, 2006 ▶); software used to prepare material for publication: *publCIF* (Westrip, 2010 ▶).

## Supplementary Material

Crystal structure: contains datablock(s) global, I. DOI: 10.1107/S1600536812016261/hb6737sup1.cif

Structure factors: contains datablock(s) I. DOI: 10.1107/S1600536812016261/hb6737Isup2.hkl

Supplementary material file. DOI: 10.1107/S1600536812016261/hb6737Isup3.cml

Additional supplementary materials:  crystallographic information; 3D view; checkCIF report

## Figures and Tables

**Table 1:** Hydrogen-bond geometry (Å, °) *Cg*1–*Cg*4 are the centroids of the C39–C44, C31–C36, C12–C17 and C4–C9 rings, respectively.

*D*—H⋯*A*	*D*—H	H⋯*A*	*D*⋯*A*	*D*—H⋯*A*
C11—H11*B*⋯*Cg*1^i^	0.98	2.97	3.777 (4)	140
C18—H18*B*⋯*Cg*2^ii^	0.98	2.91	3.688 (4)	137
C38—H38*C*⋯*Cg*3^iii^	0.98	2.84	3.756 (4)	155
C45—H45*B*⋯*Cg*4^iv^	0.98	2.77	3.596 (4)	142

Symmetry codes: (i) 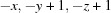; (ii) 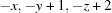; (iii) 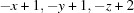; (iv) 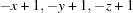.

**Fig. 1.**
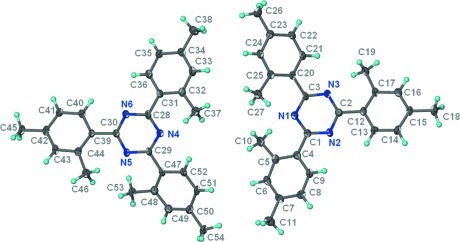
The molecular structures of the two independent molecules comprising the asymmetric unit of (I) showing displacement ellipsoids at the 70% probability level.

**Fig. 2.**
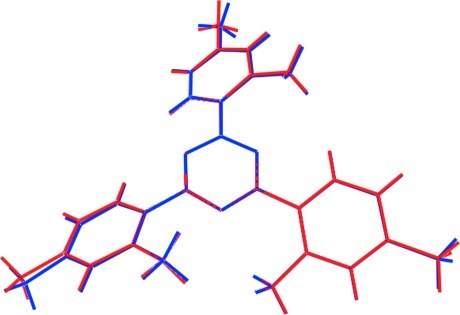
An overlay diagram of two independent molecules in (I). The N1-containing molecule is illustrated in red and the N4-molecule in blue. Molecules have been aligned so that the N1,N2,N3 and N5,N6,N4 planes are overlapped.

**Fig. 3.**
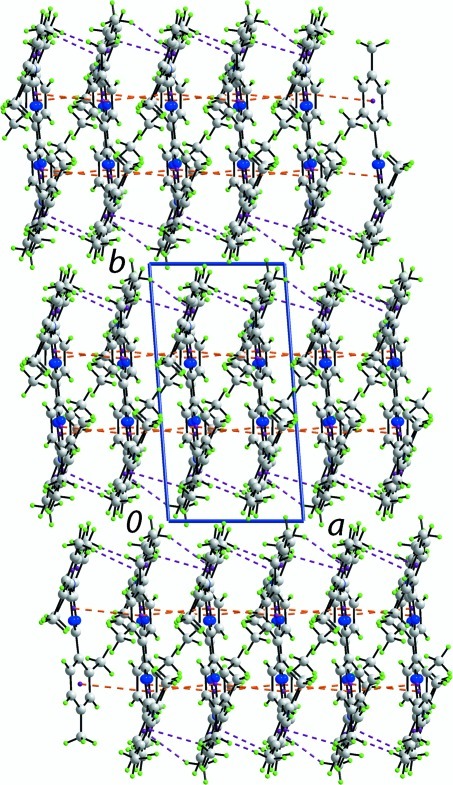
A view in projection down the c axis of the unit-cell contents for (I). The C—H···π and π–π interactions are shown as purple and orange dashed lines, respectively.

## supplementary crystallographic information

### Comment

*s*-Triphenyltriazine forms a number of adducts with metal salts; its crystal structure was reported some time ago (Damiani *et al.*, 1965). The crystal structures of substituted derivatives have also been reported, *e.g*. the *p*-tolyl (Thalladi *et al.*, 1999; Volkis *et al.*, 2003). The structure of tris(mesityl)-1,3,5-triazine is known as its silver adduct (Bosch & Barnes, 2002). Herein, the crystal and molecular structure of 2,4,6-tris(2,4-dimethylphenyl)-1,3,5-triazine (I) is described.

Two independent molecules comprise the crystallographic asymmetric unit of (I), Fig. 1. The molecules are virtually superimposable as seen from the overlay diagram, Fig. 2. With respect to the central 1,3,5-triazine ring with the N1—N3 atoms, the dihedral angles with the attached benzene rings C4—C9, C12—C17 and C20—C25 are 29.75 (15), 26.26 (15) and 21.22 (15)°, respectively. The comparable dihedral angles for the N4—N6 triazine and the C31—C36, C39—C44 and C47—C52 rings are 25.23 (15), 31.36 (14) and 20.88 (14)°, respectively. Within each molecule, one of the 2,4-dimethylphenyl residues is orientated in the opposite direction to the other two so that the molecules do not have molecular 3-fold symmetry. Overall, the shape of each molecule is of a flattened bowl.

The crystal packing is dominated by C—H···π, Table 1, and π—π interactions. The former occur between the independent molecules with each forming two donor and two acceptor interactions. The π—π interactions occur between like molecules with the shortest contacts occurring between triazine and benzene rings [inter-centroid (N1-triazine)···(C20–C25)^i^ distance = 3.7800 (18) Å, angle of inclination = 21.22 (15)° for symmetry operation *i*: 1 - *x*, 1 - *y*, 2 - *z*, and inter-centroid (N4-triazine)···(C47–C52)^ii^ distance = 3.7696 (17) Å, angle of inclination = 20.88 (14)° for symmetry operation *i*: -*x*, 1 - *y*, 2 - *z*]. The result is the formation of supramolecular layers in the *ac* plane that stack along the *b* axis with no specific interactions between them, Fig. 3.

### Experimental

The compound was synthesized by the Friedel-Crafts arylation of cyanuric chloride with excess *m*-xylene in the presence of aluminium trichloride (Orban *et al.*, 1988).

Aluminium chloride (8.7 g, 0.066 mol) was added to cyanuric chloride (4.0 g, 0.022 mol) in chlorobenzene (20 ml). The suspension was stirred by spinning a stirrer at about 250 rpm. It was then heated at 358 K for 20 min. *m*-Xylene (3.0 ml, 0.0242 mol) was added over 30 min; the reaction was exothermic. A second 3 ml portion was added over the next 30 min. The small amount of hydrogen chloride gas that was released was neutralized by 5% sodium hydroxide. A further 3 ml was added over 30 min while keeping the mixture heated at 373 K. The dark-brown reaction mixture was additionally stirred at 378 K for another 20 min.

The warm reaction mixture was added to water (30 ml). The mixture was stirred at 333 K for 10 min. The organic phase was separated and treated with 5% hydrochloric acid. This procedure was repeated.

The solvent was removed and the dark-brown residue was dried at 373 K. It was then transferred it into a 100 ml distillation flask. Toluene (50 ml) was added and the mixture heated at 348 K. To this was added ethanol (15 ml). The solution was set aside for the crystallization of the compound to give 5.4 g of a light-yellow product. The pure compound was obtained as colourless plates after recrystallization from a toluene and ethanol (5:1) mixture in a yield of 3.5 g.

### Refinement

Carbon-bound H-atoms were placed in calculated positions [C—H = 0.95 to 0.98 Å, *U*_iso_(H) 1.2 to 1.5*U*_eq_(C)] and were included in the refinement in the riding model approximation. The crystal is a non-merohedral twin with a twin component of 20.7 (3)%; the twin components were identified by the *TwinRotMat* routine in *PLATON* (Spek, 2009).

Owing to poor agreement several reflections, *i.e*. (4 2 4), (3 5 16), (4 4 6), (4 1 0), (4 3 8), (35 16), (4 4 0) and (4 2 2), were omitted from the final refinement.

### Crystal data

**Table:**

C_27_H_27_N_3_	*Z* = 4
*M**_r_* = 393.52	*F*(000) = 840
Triclinic, *P*1	*D*_x_ = 1.248 Mg m^−^^3^
Hall symbol: -P 1	Mo *K*α radiation, λ = 0.71073 Å
*a* = 7.4663 (4) Å	Cell parameters from 2806 reflections
*b* = 15.0789 (13) Å	θ = 2.7–27.5°
*c* = 19.7266 (12) Å	µ = 0.07 mm^−^^1^
α = 109.016 (7)°	*T* = 100 K
β = 90.949 (5)°	Plate, colourless
γ = 93.717 (6)°	0.30 × 0.15 × 0.05 mm
*V* = 2093.6 (2) Å^3^	

### Data collection

**Table:**

Agilent SuperNova Dual diffractometer with an Atlas detector	9605 independent reflections
Radiation source: SuperNova (Mo) X-ray Source	5518 reflections with *I* > 2σ(*I*)
Mirror monochromator	*R*_int_ = 0.058
Detector resolution: 10.4041 pixels mm^-1^	θ_max_ = 27.6°, θ_min_ = 2.7°
ω scan	*h* = −9→7
Absorption correction: multi-scan (*CrysAlis PRO*; Agilent, 2012)	*k* = −16→19
*T*_min_ = 0.978, *T*_max_ = 0.996	*l* = −25→25
14777 measured reflections	

### Refinement

**Table:**

Refinement on *F*^2^	Primary atom site location: structure-invariant direct methods
Least-squares matrix: full	Secondary atom site location: difference Fourier map
*R*[*F*^2^ > 2σ(*F*^2^)] = 0.085	Hydrogen site location: inferred from neighbouring sites
*wR*(*F*^2^) = 0.222	H-atom parameters constrained
*S* = 1.04	*w* = 1/[σ^2^(*F*_o_^2^) + (0.0673*P*)^2^ + 1.4043*P*] where *P* = (*F*_o_^2^ + 2*F*_c_^2^)/3
9597 reflections	(Δ/σ)_max_ = 0.001
554 parameters	Δρ_max_ = 0.36 e Å^−^^3^
0 restraints	Δρ_min_ = −0.39 e Å^−^^3^

### Fractional atomic coordinates and isotropic or equivalent isotropic displacement parameters (Å^2^)

**Table:**

	*x*	*y*	*z*	*U*_iso_*/*U*_eq_	
N1	0.2281 (4)	0.3830 (2)	0.89667 (14)	0.0177 (6)	
N2	0.2497 (4)	0.2425 (2)	0.92575 (14)	0.0178 (6)	
N3	0.2594 (4)	0.3902 (2)	1.01875 (14)	0.0169 (6)	
N4	0.2354 (3)	0.6128 (2)	0.57186 (14)	0.0164 (6)	
N5	0.2624 (4)	0.6141 (2)	0.45247 (14)	0.0171 (6)	
N6	0.2400 (4)	0.7575 (2)	0.54849 (14)	0.0183 (6)	
C1	0.2352 (4)	0.2892 (2)	0.87866 (17)	0.0164 (7)	
C2	0.2602 (4)	0.2959 (2)	0.99515 (17)	0.0169 (7)	
C3	0.2415 (4)	0.4304 (2)	0.96749 (17)	0.0160 (7)	
C4	0.2182 (4)	0.2313 (2)	0.80171 (18)	0.0180 (7)	
C5	0.2769 (4)	0.2636 (2)	0.74558 (18)	0.0197 (7)	
C6	0.2461 (4)	0.2028 (3)	0.67513 (18)	0.0205 (8)	
H6	0.2847	0.2238	0.6369	0.025*	
C7	0.1615 (5)	0.1129 (3)	0.65849 (18)	0.0213 (8)	
C8	0.1094 (4)	0.0810 (2)	0.71473 (18)	0.0199 (7)	
H8	0.0544	0.0193	0.7048	0.024*	
C9	0.1381 (4)	0.1396 (2)	0.78498 (18)	0.0195 (7)	
H9	0.1026	0.1171	0.8228	0.023*	
C10	0.3764 (5)	0.3579 (3)	0.75557 (18)	0.0235 (8)	
H10A	0.4585	0.3746	0.7978	0.035*	
H10B	0.2899	0.4060	0.7624	0.035*	
H10C	0.4451	0.3543	0.7130	0.035*	
C11	0.1230 (5)	0.0521 (3)	0.58162 (18)	0.0277 (9)	
H11A	0.2109	0.0696	0.5510	0.042*	
H11B	0.0018	0.0612	0.5664	0.042*	
H11C	0.1310	−0.0141	0.5774	0.042*	
C12	0.2612 (4)	0.2466 (2)	1.04930 (17)	0.0172 (7)	
C13	0.1863 (5)	0.1538 (2)	1.02738 (18)	0.0203 (8)	
H13	0.1509	0.1234	0.9783	0.024*	
C14	0.1624 (5)	0.1049 (3)	1.07584 (18)	0.0219 (8)	
H14	0.1100	0.0421	1.0597	0.026*	
C15	0.2147 (4)	0.1475 (2)	1.14763 (18)	0.0185 (7)	
C16	0.2977 (4)	0.2383 (2)	1.16825 (18)	0.0202 (8)	
H16	0.3387	0.2668	1.2169	0.024*	
C17	0.3236 (4)	0.2895 (2)	1.12107 (17)	0.0181 (7)	
C18	0.1833 (5)	0.0972 (3)	1.20146 (19)	0.0264 (8)	
H18A	0.1205	0.0354	1.1776	0.040*	
H18B	0.1101	0.1344	1.2397	0.040*	
H18C	0.2989	0.0892	1.2221	0.040*	
C19	0.4222 (5)	0.3859 (2)	1.15022 (18)	0.0229 (8)	
H19A	0.4935	0.3903	1.1935	0.034*	
H19B	0.3350	0.4340	1.1621	0.034*	
H19C	0.5018	0.3958	1.1139	0.034*	
C20	0.2397 (4)	0.5344 (2)	0.99277 (17)	0.0159 (7)	
C21	0.3143 (4)	0.5832 (2)	1.06164 (17)	0.0177 (7)	
H21	0.3577	0.5484	1.0901	0.021*	
C22	0.3266 (4)	0.6807 (2)	1.08958 (18)	0.0193 (7)	
H22	0.3776	0.7118	1.1365	0.023*	
C23	0.2634 (4)	0.7331 (2)	1.04829 (18)	0.0185 (7)	
C24	0.1862 (4)	0.6847 (2)	0.98088 (18)	0.0200 (7)	
H24	0.1413	0.7200	0.9532	0.024*	
C25	0.1710 (4)	0.5862 (2)	0.95141 (17)	0.0184 (7)	
C26	0.2836 (5)	0.8396 (3)	1.0768 (2)	0.0288 (9)	
H26A	0.2511	0.8645	1.0385	0.043*	
H26B	0.4085	0.8606	1.0933	0.043*	
H26C	0.2043	0.8625	1.1169	0.043*	
C27	0.0759 (5)	0.5441 (3)	0.87846 (18)	0.0223 (8)	
H27A	0.0037	0.5910	0.8688	0.033*	
H27B	−0.0025	0.4891	0.8777	0.033*	
H27C	0.1650	0.5249	0.8415	0.033*	
C28	0.2320 (4)	0.7064 (2)	0.59323 (17)	0.0171 (7)	
C29	0.2525 (4)	0.5694 (2)	0.50159 (17)	0.0156 (7)	
C30	0.2538 (4)	0.7078 (2)	0.47921 (17)	0.0160 (7)	
C31	0.2327 (4)	0.7581 (2)	0.67135 (17)	0.0170 (7)	
C32	0.1785 (4)	0.7160 (2)	0.72288 (18)	0.0188 (7)	
C33	0.2087 (4)	0.7689 (3)	0.79568 (18)	0.0209 (8)	
H33	0.1746	0.7408	0.8307	0.025*	
C34	0.2864 (4)	0.8609 (3)	0.81899 (18)	0.0202 (8)	
C35	0.3299 (5)	0.9030 (2)	0.76749 (18)	0.0212 (8)	
H35	0.3784	0.9665	0.7819	0.025*	
C36	0.3023 (4)	0.8519 (2)	0.69500 (18)	0.0204 (8)	
H36	0.3316	0.8816	0.6605	0.024*	
C37	0.0851 (5)	0.6187 (2)	0.70535 (18)	0.0230 (8)	
H37A	0.0180	0.6147	0.7467	0.034*	
H37B	0.1748	0.5719	0.6942	0.034*	
H37C	0.0021	0.6064	0.6638	0.034*	
C38	0.3233 (5)	0.9130 (3)	0.89758 (18)	0.0273 (9)	
H38A	0.2176	0.9048	0.9243	0.041*	
H38B	0.3500	0.9800	0.9049	0.041*	
H38C	0.4264	0.8881	0.9147	0.041*	
C39	0.2682 (4)	0.7613 (2)	0.42778 (17)	0.0155 (7)	
C40	0.3514 (4)	0.8527 (2)	0.45439 (18)	0.0184 (7)	
H40	0.3859	0.8784	0.5039	0.022*	
C41	0.3844 (4)	0.9064 (2)	0.40997 (18)	0.0193 (7)	
H41	0.4430	0.9677	0.4290	0.023*	
C42	0.3317 (4)	0.8704 (2)	0.33773 (18)	0.0191 (7)	
C43	0.2414 (4)	0.7819 (2)	0.31238 (18)	0.0195 (7)	
H43	0.1998	0.7584	0.2636	0.023*	
C44	0.2091 (4)	0.7257 (2)	0.35570 (18)	0.0175 (7)	
C45	0.3768 (5)	0.9246 (2)	0.28692 (19)	0.0218 (8)	
H45A	0.2726	0.9197	0.2547	0.033*	
H45B	0.4789	0.8982	0.2586	0.033*	
H45C	0.4079	0.9908	0.3146	0.033*	
C46	0.1078 (5)	0.6305 (2)	0.32061 (18)	0.0242 (8)	
H46A	0.0205	0.6185	0.3537	0.036*	
H46B	0.1929	0.5813	0.3089	0.036*	
H46C	0.0447	0.6302	0.2766	0.036*	
C47	0.2593 (4)	0.4660 (2)	0.47766 (17)	0.0167 (7)	
C48	0.3293 (4)	0.4124 (3)	0.41262 (18)	0.0198 (8)	
C49	0.3207 (4)	0.3143 (3)	0.39598 (18)	0.0203 (8)	
H49	0.3674	0.2778	0.3519	0.024*	
C50	0.2477 (4)	0.2679 (2)	0.44048 (19)	0.0200 (7)	
C51	0.1841 (4)	0.3229 (2)	0.50602 (18)	0.0203 (8)	
H51	0.1363	0.2935	0.5383	0.024*	
C52	0.1902 (4)	0.4195 (2)	0.52416 (17)	0.0173 (7)	
H52	0.1469	0.4555	0.5690	0.021*	
C53	0.4187 (5)	0.4523 (3)	0.35912 (18)	0.0219 (8)	
H53A	0.4951	0.4061	0.3287	0.033*	
H53B	0.4924	0.5101	0.3850	0.033*	
H53C	0.3264	0.4666	0.3291	0.033*	
C54	0.2342 (5)	0.1619 (2)	0.4188 (2)	0.0269 (8)	
H54A	0.2463	0.1431	0.4616	0.040*	
H54B	0.3303	0.1369	0.3863	0.040*	
H54C	0.1174	0.1369	0.3944	0.040*	

### Atomic displacement parameters (Å^2^)

**Table:**

	*U*^11^	*U*^22^	*U*^33^	*U*^12^	*U*^13^	*U*^23^
N1	0.0190 (15)	0.0186 (16)	0.0168 (15)	0.0022 (11)	0.0005 (11)	0.0072 (12)
N2	0.0189 (15)	0.0212 (16)	0.0149 (15)	−0.0009 (11)	−0.0018 (11)	0.0088 (13)
N3	0.0200 (15)	0.0170 (15)	0.0151 (15)	−0.0002 (11)	0.0005 (11)	0.0075 (12)
N4	0.0181 (15)	0.0171 (15)	0.0148 (15)	0.0004 (11)	0.0002 (11)	0.0065 (12)
N5	0.0203 (15)	0.0178 (15)	0.0159 (15)	0.0018 (11)	0.0017 (11)	0.0091 (12)
N6	0.0215 (15)	0.0194 (16)	0.0151 (15)	−0.0016 (12)	0.0004 (11)	0.0078 (12)
C1	0.0154 (17)	0.0183 (18)	0.0160 (17)	0.0022 (13)	0.0027 (12)	0.0061 (14)
C2	0.0159 (17)	0.0190 (18)	0.0158 (17)	0.0002 (13)	0.0007 (12)	0.0059 (14)
C3	0.0151 (17)	0.0221 (19)	0.0129 (17)	0.0009 (13)	0.0002 (12)	0.0088 (14)
C4	0.0179 (17)	0.0206 (19)	0.0168 (17)	0.0042 (13)	0.0003 (12)	0.0073 (15)
C5	0.0195 (18)	0.0221 (19)	0.0200 (18)	0.0018 (14)	−0.0001 (13)	0.0101 (15)
C6	0.0195 (18)	0.028 (2)	0.0162 (18)	0.0071 (14)	0.0010 (13)	0.0100 (16)
C7	0.0214 (19)	0.027 (2)	0.0150 (18)	0.0065 (14)	0.0000 (13)	0.0048 (15)
C8	0.0174 (18)	0.0193 (19)	0.0227 (19)	0.0026 (13)	−0.0002 (13)	0.0062 (15)
C9	0.0203 (18)	0.0215 (19)	0.0196 (18)	0.0010 (14)	0.0008 (13)	0.0108 (15)
C10	0.028 (2)	0.027 (2)	0.0194 (19)	0.0045 (15)	0.0058 (14)	0.0115 (16)
C11	0.025 (2)	0.033 (2)	0.022 (2)	−0.0002 (16)	−0.0011 (14)	0.0046 (17)
C12	0.0183 (17)	0.0188 (18)	0.0178 (18)	0.0052 (13)	0.0022 (12)	0.0098 (15)
C13	0.0250 (19)	0.0174 (19)	0.0178 (18)	−0.0001 (14)	0.0005 (14)	0.0050 (15)
C14	0.027 (2)	0.0180 (19)	0.024 (2)	0.0019 (14)	0.0023 (14)	0.0117 (16)
C15	0.0186 (18)	0.0202 (19)	0.0214 (19)	0.0046 (13)	0.0037 (13)	0.0124 (15)
C16	0.0230 (19)	0.022 (2)	0.0161 (18)	0.0034 (14)	−0.0002 (13)	0.0071 (15)
C17	0.0179 (17)	0.0230 (19)	0.0156 (17)	0.0019 (13)	0.0019 (12)	0.0093 (15)
C18	0.031 (2)	0.029 (2)	0.025 (2)	0.0021 (16)	0.0009 (15)	0.0173 (17)
C19	0.029 (2)	0.026 (2)	0.0151 (18)	−0.0032 (15)	−0.0022 (14)	0.0086 (16)
C20	0.0164 (17)	0.0178 (18)	0.0158 (17)	0.0020 (13)	0.0018 (12)	0.0085 (14)
C21	0.0177 (17)	0.0222 (19)	0.0159 (17)	−0.0002 (13)	−0.0010 (12)	0.0104 (15)
C22	0.0220 (18)	0.0214 (19)	0.0141 (17)	0.0015 (14)	0.0001 (13)	0.0053 (15)
C23	0.0172 (17)	0.0188 (18)	0.0214 (19)	0.0019 (13)	0.0044 (13)	0.0090 (15)
C24	0.0242 (19)	0.022 (2)	0.0166 (18)	0.0059 (14)	0.0041 (13)	0.0098 (15)
C25	0.0191 (18)	0.0208 (19)	0.0175 (18)	0.0030 (13)	0.0026 (13)	0.0088 (15)
C26	0.041 (2)	0.020 (2)	0.026 (2)	0.0015 (16)	0.0005 (16)	0.0093 (17)
C27	0.0255 (19)	0.025 (2)	0.0186 (18)	0.0052 (15)	0.0018 (14)	0.0091 (16)
C28	0.0168 (17)	0.0217 (19)	0.0164 (17)	−0.0005 (13)	0.0011 (12)	0.0114 (15)
C29	0.0163 (17)	0.0191 (18)	0.0134 (17)	−0.0006 (13)	−0.0007 (12)	0.0087 (14)
C30	0.0173 (17)	0.0181 (18)	0.0140 (17)	−0.0005 (13)	0.0007 (12)	0.0076 (14)
C31	0.0192 (17)	0.0186 (18)	0.0145 (17)	0.0020 (13)	−0.0002 (12)	0.0074 (14)
C32	0.0185 (18)	0.0213 (19)	0.0194 (18)	0.0014 (14)	−0.0018 (13)	0.0107 (15)
C33	0.0227 (19)	0.025 (2)	0.0186 (18)	0.0003 (14)	0.0018 (13)	0.0119 (16)
C34	0.0203 (18)	0.024 (2)	0.0162 (18)	0.0029 (14)	0.0016 (13)	0.0062 (15)
C35	0.0260 (19)	0.0165 (18)	0.0218 (19)	0.0002 (14)	0.0017 (14)	0.0077 (15)
C36	0.0244 (19)	0.023 (2)	0.0164 (18)	0.0019 (14)	0.0041 (13)	0.0095 (15)
C37	0.0251 (19)	0.023 (2)	0.0220 (19)	−0.0058 (15)	0.0020 (14)	0.0099 (16)
C38	0.032 (2)	0.031 (2)	0.0171 (19)	0.0004 (16)	0.0031 (15)	0.0059 (17)
C39	0.0180 (17)	0.0158 (17)	0.0152 (17)	0.0017 (13)	0.0035 (12)	0.0082 (14)
C40	0.0199 (18)	0.0210 (19)	0.0159 (17)	0.0017 (14)	0.0016 (13)	0.0081 (15)
C41	0.0218 (18)	0.0171 (18)	0.0205 (18)	−0.0005 (13)	0.0020 (13)	0.0087 (15)
C42	0.0193 (18)	0.0208 (19)	0.0223 (19)	0.0045 (13)	0.0060 (13)	0.0131 (16)
C43	0.0181 (17)	0.027 (2)	0.0188 (18)	0.0031 (14)	0.0032 (13)	0.0139 (16)
C44	0.0180 (17)	0.0181 (18)	0.0185 (18)	−0.0002 (13)	0.0035 (13)	0.0087 (15)
C45	0.0266 (19)	0.0191 (19)	0.025 (2)	0.0012 (14)	0.0038 (14)	0.0139 (16)
C46	0.029 (2)	0.023 (2)	0.0215 (19)	−0.0018 (15)	−0.0043 (14)	0.0097 (16)
C47	0.0156 (17)	0.0231 (19)	0.0147 (17)	0.0012 (13)	0.0005 (12)	0.0108 (15)
C48	0.0187 (18)	0.0224 (19)	0.0206 (19)	−0.0007 (14)	−0.0021 (13)	0.0105 (15)
C49	0.0213 (18)	0.0219 (19)	0.0188 (18)	0.0044 (14)	0.0003 (13)	0.0076 (15)
C50	0.0208 (18)	0.0185 (18)	0.0229 (19)	−0.0013 (14)	−0.0022 (13)	0.0103 (16)
C51	0.0234 (19)	0.0200 (19)	0.0227 (19)	0.0000 (14)	0.0022 (14)	0.0144 (16)
C52	0.0187 (18)	0.0202 (18)	0.0150 (17)	0.0001 (13)	0.0007 (13)	0.0086 (15)
C53	0.0214 (19)	0.026 (2)	0.0218 (19)	0.0024 (14)	0.0015 (14)	0.0117 (16)
C54	0.038 (2)	0.018 (2)	0.026 (2)	0.0004 (16)	0.0031 (16)	0.0110 (17)

### Geometric parameters (Å, º)

**Table:**

N1—C3	1.345 (4)	C26—H26A	0.9800
N1—C1	1.346 (4)	C26—H26B	0.9800
N2—C2	1.341 (4)	C26—H26C	0.9800
N2—C1	1.343 (4)	C27—H27A	0.9800
N3—C2	1.344 (4)	C27—H27B	0.9800
N3—C3	1.347 (4)	C27—H27C	0.9800
N4—C28	1.338 (4)	C28—C31	1.483 (5)
N4—C29	1.338 (4)	C29—C47	1.478 (5)
N5—C30	1.344 (4)	C30—C39	1.489 (4)
N5—C29	1.350 (4)	C31—C36	1.398 (5)
N6—C30	1.337 (4)	C31—C32	1.415 (4)
N6—C28	1.347 (4)	C32—C33	1.403 (5)
C1—C4	1.483 (5)	C32—C37	1.512 (4)
C2—C12	1.487 (5)	C33—C34	1.394 (5)
C3—C20	1.484 (5)	C33—H33	0.9500
C4—C9	1.403 (5)	C34—C35	1.395 (5)
C4—C5	1.412 (5)	C34—C38	1.504 (5)
C5—C6	1.399 (5)	C35—C36	1.390 (5)
C5—C10	1.513 (5)	C35—H35	0.9500
C6—C7	1.391 (5)	C36—H36	0.9500
C6—H6	0.9500	C37—H37A	0.9800
C7—C8	1.397 (5)	C37—H37B	0.9800
C7—C11	1.506 (5)	C37—H37C	0.9800
C8—C9	1.383 (5)	C38—H38A	0.9800
C8—H8	0.9500	C38—H38B	0.9800
C9—H9	0.9500	C38—H38C	0.9800
C10—H10A	0.9800	C39—C44	1.399 (4)
C10—H10B	0.9800	C39—C40	1.403 (4)
C10—H10C	0.9800	C40—C41	1.388 (5)
C11—H11A	0.9800	C40—H40	0.9500
C11—H11B	0.9800	C41—C42	1.389 (5)
C11—H11C	0.9800	C41—H41	0.9500
C12—C13	1.398 (4)	C42—C43	1.388 (5)
C12—C17	1.408 (4)	C42—C45	1.514 (4)
C13—C14	1.392 (5)	C43—C44	1.399 (4)
C13—H13	0.9500	C43—H43	0.9500
C14—C15	1.388 (5)	C44—C46	1.519 (4)
C14—H14	0.9500	C45—H45A	0.9800
C15—C16	1.393 (5)	C45—H45B	0.9800
C15—C18	1.506 (5)	C45—H45C	0.9800
C16—C17	1.398 (5)	C46—H46A	0.9800
C16—H16	0.9500	C46—H46B	0.9800
C17—C19	1.513 (5)	C46—H46C	0.9800
C18—H18A	0.9800	C47—C48	1.405 (5)
C18—H18B	0.9800	C47—C52	1.409 (4)
C18—H18C	0.9800	C48—C49	1.403 (5)
C19—H19A	0.9800	C48—C53	1.519 (5)
C19—H19B	0.9800	C49—C50	1.387 (5)
C19—H19C	0.9800	C49—H49	0.9500
C20—C21	1.402 (4)	C50—C51	1.399 (5)
C20—C25	1.411 (5)	C50—C54	1.510 (5)
C21—C22	1.389 (5)	C51—C52	1.379 (5)
C21—H21	0.9500	C51—H51	0.9500
C22—C23	1.401 (5)	C52—H52	0.9500
C22—H22	0.9500	C53—H53A	0.9800
C23—C24	1.386 (5)	C53—H53B	0.9800
C23—C26	1.515 (5)	C53—H53C	0.9800
C24—C25	1.404 (5)	C54—H54A	0.9800
C24—H24	0.9500	C54—H54B	0.9800
C25—C27	1.514 (4)	C54—H54C	0.9800
			
C3—N1—C1	115.2 (3)	C25—C27—H27C	109.5
C2—N2—C1	115.5 (3)	H27A—C27—H27C	109.5
C2—N3—C3	115.6 (3)	H27B—C27—H27C	109.5
C28—N4—C29	116.5 (3)	N4—C28—N6	124.0 (3)
C30—N5—C29	114.8 (3)	N4—C28—C31	118.2 (3)
C30—N6—C28	115.1 (3)	N6—C28—C31	117.6 (3)
N2—C1—N1	124.8 (3)	N4—C29—N5	124.0 (3)
N2—C1—C4	116.5 (3)	N4—C29—C47	116.9 (3)
N1—C1—C4	118.7 (3)	N5—C29—C47	119.1 (3)
N2—C2—N3	124.4 (3)	N6—C30—N5	125.5 (3)
N2—C2—C12	117.3 (3)	N6—C30—C39	117.2 (3)
N3—C2—C12	118.1 (3)	N5—C30—C39	117.3 (3)
N1—C3—N3	124.5 (3)	C36—C31—C32	118.8 (3)
N1—C3—C20	119.3 (3)	C36—C31—C28	117.5 (3)
N3—C3—C20	116.2 (3)	C32—C31—C28	123.6 (3)
C9—C4—C5	119.2 (3)	C33—C32—C31	117.9 (3)
C9—C4—C1	117.0 (3)	C33—C32—C37	117.3 (3)
C5—C4—C1	123.7 (3)	C31—C32—C37	124.7 (3)
C6—C5—C4	117.8 (3)	C34—C33—C32	123.0 (3)
C6—C5—C10	117.1 (3)	C34—C33—H33	118.5
C4—C5—C10	125.1 (3)	C32—C33—H33	118.5
C7—C6—C5	123.0 (3)	C33—C34—C35	118.2 (3)
C7—C6—H6	118.5	C33—C34—C38	120.8 (3)
C5—C6—H6	118.5	C35—C34—C38	121.0 (3)
C6—C7—C8	118.5 (3)	C36—C35—C34	119.9 (3)
C6—C7—C11	120.8 (3)	C36—C35—H35	120.0
C8—C7—C11	120.7 (3)	C34—C35—H35	120.0
C9—C8—C7	119.9 (3)	C35—C36—C31	122.0 (3)
C9—C8—H8	120.1	C35—C36—H36	119.0
C7—C8—H8	120.1	C31—C36—H36	119.0
C8—C9—C4	121.6 (3)	C32—C37—H37A	109.5
C8—C9—H9	119.2	C32—C37—H37B	109.5
C4—C9—H9	119.2	H37A—C37—H37B	109.5
C5—C10—H10A	109.5	C32—C37—H37C	109.5
C5—C10—H10B	109.5	H37A—C37—H37C	109.5
H10A—C10—H10B	109.5	H37B—C37—H37C	109.5
C5—C10—H10C	109.5	C34—C38—H38A	109.5
H10A—C10—H10C	109.5	C34—C38—H38B	109.5
H10B—C10—H10C	109.5	H38A—C38—H38B	109.5
C7—C11—H11A	109.5	C34—C38—H38C	109.5
C7—C11—H11B	109.5	H38A—C38—H38C	109.5
H11A—C11—H11B	109.5	H38B—C38—H38C	109.5
C7—C11—H11C	109.5	C44—C39—C40	118.9 (3)
H11A—C11—H11C	109.5	C44—C39—C30	124.6 (3)
H11B—C11—H11C	109.5	C40—C39—C30	116.5 (3)
C13—C12—C17	119.1 (3)	C41—C40—C39	121.5 (3)
C13—C12—C2	116.9 (3)	C41—C40—H40	119.3
C17—C12—C2	123.9 (3)	C39—C40—H40	119.3
C14—C13—C12	121.4 (3)	C40—C41—C42	120.0 (3)
C14—C13—H13	119.3	C40—C41—H41	120.0
C12—C13—H13	119.3	C42—C41—H41	120.0
C15—C14—C13	120.3 (3)	C43—C42—C41	118.4 (3)
C15—C14—H14	119.9	C43—C42—C45	120.4 (3)
C13—C14—H14	119.8	C41—C42—C45	121.2 (3)
C14—C15—C16	117.8 (3)	C42—C43—C44	122.7 (3)
C14—C15—C18	121.1 (3)	C42—C43—H43	118.6
C16—C15—C18	121.1 (3)	C44—C43—H43	118.6
C15—C16—C17	123.4 (3)	C39—C44—C43	118.4 (3)
C15—C16—H16	118.3	C39—C44—C46	124.8 (3)
C17—C16—H16	118.3	C43—C44—C46	116.8 (3)
C16—C17—C12	117.8 (3)	C42—C45—H45A	109.5
C16—C17—C19	117.6 (3)	C42—C45—H45B	109.5
C12—C17—C19	124.6 (3)	H45A—C45—H45B	109.5
C15—C18—H18A	109.5	C42—C45—H45C	109.5
C15—C18—H18B	109.5	H45A—C45—H45C	109.5
H18A—C18—H18B	109.5	H45B—C45—H45C	109.5
C15—C18—H18C	109.5	C44—C46—H46A	109.5
H18A—C18—H18C	109.5	C44—C46—H46B	109.5
H18B—C18—H18C	109.5	H46A—C46—H46B	109.5
C17—C19—H19A	109.5	C44—C46—H46C	109.5
C17—C19—H19B	109.5	H46A—C46—H46C	109.5
H19A—C19—H19B	109.5	H46B—C46—H46C	109.5
C17—C19—H19C	109.5	C48—C47—C52	118.8 (3)
H19A—C19—H19C	109.5	C48—C47—C29	124.5 (3)
H19B—C19—H19C	109.5	C52—C47—C29	116.7 (3)
C21—C20—C25	118.8 (3)	C49—C48—C47	118.0 (3)
C21—C20—C3	117.1 (3)	C49—C48—C53	116.8 (3)
C25—C20—C3	124.0 (3)	C47—C48—C53	125.1 (3)
C22—C21—C20	122.1 (3)	C50—C49—C48	123.4 (3)
C22—C21—H21	119.0	C50—C49—H49	118.3
C20—C21—H21	119.0	C48—C49—H49	118.3
C21—C22—C23	119.7 (3)	C49—C50—C51	117.6 (3)
C21—C22—H22	120.2	C49—C50—C54	121.5 (3)
C23—C22—H22	120.2	C51—C50—C54	121.0 (3)
C24—C23—C22	118.2 (3)	C52—C51—C50	120.6 (3)
C24—C23—C26	121.6 (3)	C52—C51—H51	119.7
C22—C23—C26	120.2 (3)	C50—C51—H51	119.7
C23—C24—C25	123.4 (3)	C51—C52—C47	121.5 (3)
C23—C24—H24	118.3	C51—C52—H52	119.2
C25—C24—H24	118.3	C47—C52—H52	119.2
C24—C25—C20	117.8 (3)	C48—C53—H53A	109.5
C24—C25—C27	116.9 (3)	C48—C53—H53B	109.5
C20—C25—C27	125.3 (3)	H53A—C53—H53B	109.5
C23—C26—H26A	109.5	C48—C53—H53C	109.5
C23—C26—H26B	109.5	H53A—C53—H53C	109.5
H26A—C26—H26B	109.5	H53B—C53—H53C	109.5
C23—C26—H26C	109.5	C50—C54—H54A	109.5
H26A—C26—H26C	109.5	C50—C54—H54B	109.5
H26B—C26—H26C	109.5	H54A—C54—H54B	109.5
C25—C27—H27A	109.5	C50—C54—H54C	109.5
C25—C27—H27B	109.5	H54A—C54—H54C	109.5
H27A—C27—H27B	109.5	H54B—C54—H54C	109.5
			
C2—N2—C1—N1	−1.1 (5)	C29—N4—C28—N6	1.7 (5)
C2—N2—C1—C4	−178.3 (3)	C29—N4—C28—C31	−173.6 (3)
C3—N1—C1—N2	1.7 (5)	C30—N6—C28—N4	−0.6 (5)
C3—N1—C1—C4	178.9 (3)	C30—N6—C28—C31	174.8 (3)
C1—N2—C2—N3	−0.9 (5)	C28—N4—C29—N5	−1.4 (5)
C1—N2—C2—C12	175.1 (3)	C28—N4—C29—C47	179.1 (3)
C3—N3—C2—N2	1.9 (5)	C30—N5—C29—N4	0.0 (4)
C3—N3—C2—C12	−174.0 (3)	C30—N5—C29—C47	179.5 (3)
C1—N1—C3—N3	−0.5 (5)	C28—N6—C30—N5	−1.0 (5)
C1—N1—C3—C20	178.5 (3)	C28—N6—C30—C39	−178.4 (3)
C2—N3—C3—N1	−1.1 (5)	C29—N5—C30—N6	1.2 (5)
C2—N3—C3—C20	179.8 (3)	C29—N5—C30—C39	178.6 (3)
N2—C1—C4—C9	28.5 (4)	N4—C28—C31—C36	154.1 (3)
N1—C1—C4—C9	−148.9 (3)	N6—C28—C31—C36	−21.6 (5)
N2—C1—C4—C5	−151.8 (3)	N4—C28—C31—C32	−21.5 (5)
N1—C1—C4—C5	30.8 (5)	N6—C28—C31—C32	162.8 (3)
C9—C4—C5—C6	2.2 (5)	C36—C31—C32—C33	−4.2 (5)
C1—C4—C5—C6	−177.5 (3)	C28—C31—C32—C33	171.3 (3)
C9—C4—C5—C10	−175.7 (3)	C36—C31—C32—C37	174.0 (3)
C1—C4—C5—C10	4.6 (5)	C28—C31—C32—C37	−10.5 (5)
C4—C5—C6—C7	−0.2 (5)	C31—C32—C33—C34	1.1 (5)
C10—C5—C6—C7	177.9 (3)	C37—C32—C33—C34	−177.2 (3)
C5—C6—C7—C8	−1.8 (5)	C32—C33—C34—C35	2.4 (5)
C5—C6—C7—C11	176.8 (3)	C32—C33—C34—C38	−177.3 (3)
C6—C7—C8—C9	1.7 (5)	C33—C34—C35—C36	−2.6 (5)
C11—C7—C8—C9	−176.8 (3)	C38—C34—C35—C36	177.0 (3)
C7—C8—C9—C4	0.3 (5)	C34—C35—C36—C31	−0.5 (5)
C5—C4—C9—C8	−2.3 (5)	C32—C31—C36—C35	4.0 (5)
C1—C4—C9—C8	177.5 (3)	C28—C31—C36—C35	−171.8 (3)
N2—C2—C12—C13	−23.1 (4)	N6—C30—C39—C44	−151.5 (3)
N3—C2—C12—C13	153.1 (3)	N5—C30—C39—C44	30.9 (5)
N2—C2—C12—C17	160.4 (3)	N6—C30—C39—C40	30.1 (4)
N3—C2—C12—C17	−23.3 (5)	N5—C30—C39—C40	−147.5 (3)
C17—C12—C13—C14	3.9 (5)	C44—C39—C40—C41	−3.4 (5)
C2—C12—C13—C14	−172.8 (3)	C30—C39—C40—C41	175.1 (3)
C12—C13—C14—C15	−0.7 (5)	C39—C40—C41—C42	1.2 (5)
C13—C14—C15—C16	−2.6 (5)	C40—C41—C42—C43	2.1 (5)
C13—C14—C15—C18	177.5 (3)	C40—C41—C42—C45	−175.9 (3)
C14—C15—C16—C17	2.8 (5)	C41—C42—C43—C44	−3.2 (5)
C18—C15—C16—C17	−177.4 (3)	C45—C42—C43—C44	174.8 (3)
C15—C16—C17—C12	0.4 (5)	C40—C39—C44—C43	2.3 (5)
C15—C16—C17—C19	−177.7 (3)	C30—C39—C44—C43	−176.1 (3)
C13—C12—C17—C16	−3.6 (5)	C40—C39—C44—C46	−176.4 (3)
C2—C12—C17—C16	172.7 (3)	C30—C39—C44—C46	5.2 (5)
C13—C12—C17—C19	174.3 (3)	C42—C43—C44—C39	1.0 (5)
C2—C12—C17—C19	−9.3 (5)	C42—C43—C44—C46	179.8 (3)
N1—C3—C20—C21	−158.5 (3)	N4—C29—C47—C48	−159.9 (3)
N3—C3—C20—C21	20.7 (4)	N5—C29—C47—C48	20.6 (5)
N1—C3—C20—C25	20.3 (5)	N4—C29—C47—C52	20.1 (4)
N3—C3—C20—C25	−160.5 (3)	N5—C29—C47—C52	−159.5 (3)
C25—C20—C21—C22	−1.6 (5)	C52—C47—C48—C49	2.3 (5)
C3—C20—C21—C22	177.2 (3)	C29—C47—C48—C49	−177.7 (3)
C20—C21—C22—C23	−0.1 (5)	C52—C47—C48—C53	−176.0 (3)
C21—C22—C23—C24	1.5 (5)	C29—C47—C48—C53	3.9 (5)
C21—C22—C23—C26	−177.2 (3)	C47—C48—C49—C50	−0.3 (5)
C22—C23—C24—C25	−1.2 (5)	C53—C48—C49—C50	178.2 (3)
C26—C23—C24—C25	177.5 (3)	C48—C49—C50—C51	−1.7 (5)
C23—C24—C25—C20	−0.6 (5)	C48—C49—C50—C54	177.3 (3)
C23—C24—C25—C27	177.4 (3)	C49—C50—C51—C52	1.7 (5)
C21—C20—C25—C24	1.9 (5)	C54—C50—C51—C52	−177.3 (3)
C3—C20—C25—C24	−176.9 (3)	C50—C51—C52—C47	0.3 (5)
C21—C20—C25—C27	−175.8 (3)	C48—C47—C52—C51	−2.4 (5)
C3—C20—C25—C27	5.4 (5)	C29—C47—C52—C51	177.7 (3)

### Hydrogen-bond geometry (Å, º)

Cg1–Cg4 are the centroids of the C39–C44, C31–C36, C12–C17 and C4–C9 rings, respectively.

**Table:**

*D*—H···*A*	*D*—H	H···*A*	*D*···*A*	*D*—H···*A*
C11—H11*B*···*Cg*1^i^	0.98	2.97	3.777 (4)	140
C18—H18*B*···*Cg*2^ii^	0.98	2.91	3.688 (4)	137
C38—H38*C*···*Cg*3^iii^	0.98	2.84	3.756 (4)	155
C45—H45*B*···*Cg*4^iv^	0.98	2.77	3.596 (4)	142

Symmetry codes: (i) −*x*, −*y*+1, −*z*+1; (ii) −*x*, −*y*+1, −*z*+2; (iii) −*x*+1, −*y*+1, −*z*+2; (iv) −*x*+1, −*y*+1, −*z*+1.
